# Newspapers vs. the Doctors

**Published:** 1893-11

**Authors:** Henry Ryder-Taylor

**Affiliations:** San Antonio (Tex.) “Daily Light”


For Texas Medical Journal.

### NEWSPAPERS VS. THE DOCTORS.

BY HENRY RIDER-TAYLOR, OF THE SAN ANTONIO (TEX.) "DAILY LIGHT."

IN THE JULY ISSUE of THE TEXAS MEDICAL JOURNAL I found a very able and interesting editorial upon the above subject. While in some cases I agree with the writer, there are other material points on which I differ from him. As there are two sides to every question, and the question is one of great importance to both professions, I propose to place before the medical profession the newspaper men's views, in order that it may be the better understood. As I have had thirty years' experience in this country, and in England, as an editor, reporter, and journalist, I have associated much with medical men, and have discussed the subject considerably, I think I may, without egotism, venture to adopt this course.

In the first place, I am compelled to take issue with the writer when he states that the non-professional press is, as a rule, inimical to the regular medical profession. This certainly is not true, for it has done all in its power, voluntarily, and without price, in advancing the interests of worthy practitioners, and of making them famous, or popular, and thereby adding to their prestige and wealth. I am also compelled to refute the idea that because doctors do not advertise, the press is prejudiced against them. This is disproved by the fact that those doctors who get complimentary notices give no more advertising than those who do not. I also resent the inference that the press fosters quackery, because I know that the press has done more to expose quackery than all the medical associations of the country combined, for they talk literally to themselves, and the newspapers talk to their thousands of readers. I write of the press, as a rule, for there may be newspaper men who, like some doctors, are foolish and even venal.

But the great question at issue is, should doctors advertise in the newspapers? Many do and many do not. Naturally, as a newspaper man, I say "yes," for I can see no reason why doctors should not advertise, and derive the benefits of it, any more than the members of any other professions, but I certainly would have them advertise in a gentlemanly and professional way, confining their advertisement to name, degree, or titles, the college, or society, conferring them, the professional positions they have filled, or are filling, the "special" line they have, if any, and the places and hours of attendance. I would object to a "quack" advertisment from a professional as much as a board of censors, but I am quite sure that none could reasonably, or logically, object to this kind of advertising.

The cry that doctors should not advertise, is inconsistent, and even the professed objectors do it, but in an indirect way. On all doctors' residences, and at the drug stores, are found the doctors' signs; their names are capitalized in the directory (paid for as advertising), and appear on the fly leaves of hotel registers, or on cards, or banners giving hotel rules that are displayed in the guests' rooms. What are these but advertising, and would they be more out of place in a newspaper than thus publicly displayed? I think not, and I believe that most men will agree in this opinion.

It is not usual here, I admit, but I think that every doctor should not only give his degrees, but the college at which they were acquired; also his record, i. e., of positions filled, for the mutual interests of himself and his would-be patients. This is done in England, the doctor signing M. D., Lond., Edin., or other abbreviation, flanked by his F. R. C. P., or his M. R. C. S., which shows distinctly what he is and where he stands. There is, I think, good reason for this, and I will tell you why. Say, "Dr. Smith" and "Dr. Jones" arrive in a city, and propose to locate. "Dr. Smith" is a graduate, with honor, at one of the leading schools of medicine; has filled several good positions most satisfactorily, is in every way qualified, and entitled to public confidence and support. "Dr. Jones" is a beardless youth, an impractical non-entity from a doubtful, though recognized, college, full of vague theories, and devoid of practical knowledge, yet both are doctors, and the people, not knowing the material difference, would as soon have one doctor as the other. Where, then, is the reward for the experienced man, and why should an inexperienced man be foisted on the people when it may be a matter of life and death to the patient? It may be all very well to talk of "every man must have a beginning," but I rather suspect that most men—if they knew it—would object to being made the subject of experiment by an unsophisticated doctor. But how in the world are the people to know which is which, except by judicious advertising?

Now, in my experience, especially in this country, I have seen many doctors so scared by a so-called code of ethics, or so mean that they will, hypocritically, hold up their hands in pious awe at the doctor who honestly advertises in the newspapers and pays for his advertisement. and yet they will sneak around, flatter and treat a reporter for the mere purpose of getting a free "ad." of this or that case. Of course, these are comparatively few and they derive very little benefit from their meanness, for the average reporter is not caught by mere flattery, sandwiched with the drinks and cigars.

In regard to complimentary notices that appear in the newspapers respecting doctors I see no valid reasons why they, like other people, should not be subject to fair criticism and be praised or censured as the occasions demand. Law is considered as honorable a profession as that of medicine, and yet none would kick or cite a "brother" before his bar association because a paper said that "Attorney Williams" made a most eloquent and pathetic defence of his client, or that "Attorney Brown" then skillfully cross-examined Mandy Smith and elicited some important points against the plaintiff. If this be the case, as it surely is, why then should not the doctor have due credit for this treatment, that operation, etc. Why should he not receive the reward of his study and experience when that reward means the fame and wealth that he has honestly and honorably earned?

When Grover Cleveland had the tooth-ache, Mrs. Cleveland had a baby, Bismarck had a return of the gout or when any prominent person is sick, the news is flashed all over the country, the doctor's name is mentioned and even his statements are published, and no one ever thinks of kicking at the alleged violation of the code of ethics, but let some prominent citizen in a village or city be seriously ill and the doctor's name is mentioned or his statements are published and jealous rivals will howl at the violation of the code, cry "quackery" and use other undignified epithets, yet news of that sick man may be of more importance to the community than that of all the politicians and throned monarchs of the earth. Not only this, but some doctors have been cited to appear before local medical associations to answer the charge of the violation of the code of ethics, the sole ground for such charge being that their names have appeared in the newspapers in connection with their cases, and, as has been proved, without their knowledge and consent! Who were the kickers? Was it those doctors who have controlled or do control the leading practices of that locality? Oh dear, No! These men are too great for such petty meanness. * * * *

It is the duty of the newspaper man to collect all news of a legitimate character, and in this capacity he is a frequent visitor to the local doctors. Usually he is received with courtesy and all information that can be legitimately given, is accorded; but there are doctors, and the smallest in calibre is the worse offender, who churlishly refuse information on the ground that they have not time to talk and yet can be seen swilling beer or liquor by the hour in a neighboring saloon. These doctors are wise not to tell what they do not know, but they were foolish in supposing that an experienced newspaper man can be fooled in that way. And yet these men have the audacity to growl because they do not get the newspaper notices that their courteous and meritorious fellow-doctors cordially receive.

There is another phase right here that should be considered. The newspaper represents the public who naturally look upon it as a source of information and inter-communion. Now, suppose some accident occurs, or some crime is committed; where does the public seek its information? In the newspapers, and it is to the interests of all that correct data be given. Who can give authoritive information as to the nature of injuries and their probable result? Only the doctor in attendance. Then should he not, as a matter of courtesy to the public, as well as to the newspapers, give such information as he can, providing no confidence is betrayed, and the ends of justice are not frustrated? Such details are usually given, but there are doctors,—thank goodness they are few—who cannot or will not give the required facts. I remember some time since, a woman was shot, and I was detailed to investigate the case. I learned that Dr. ———— was in attendance, and spent half a day hunting him to get accurate details. At last I found him. He knew who I was, and I courteousy made my request, but he refused to state the injuries or prognosticate the result of his operation (under which the woman died, by the bye). Naturally I asked why, and he simply answered, "Because I do not choose to do so, sir." I had to get the required details as best I could, through a faulty source, and as a result, the report of injuries was slightly erroneous; then bless me, if that very doctor had not the cheek to read that report to several doctors assembled in a beer saloon and to sneeringly remark "what d—— fools these reporters are." If that doctor had done his duty as faithfully as the reporter, or had possessed a due sense of gentlemanly courtesy, the error would not have occurred. It is such men as these that antagonize the press, and then kick because the independent press will not fawn upon or flatter them.

I deny that the press fosters quackery, though it is a natural adjunct to their business. The columns of the newspapers are open to the legitimate doctor and the "quack" alike, and if either offer an advertisement the same rate is charged and the "ad." accepted, for a paper to be successful must be run on business principles, and advertising is its main source of revenue. The "quack," realizing the advantage of advertising, is a liberal advertiser, and usually a prompt payer. It is right here the quack gets in his work. He strikes a city and has a big "ad." in all papers, consisting of self-laudation, exhibition of titles that are of a bogus character, and testimonials of cures that have been secured from the deluded and mercenary. He rakes in the dollars in abundance, while the ordinary doctor drags out a bare existence. The local doctor has only himself to blame. Why? Simply this, because few know him, and even those who have heard of him are not acquinted with those qualifications that would, if known, have commanded a lucrative support. I am satisfied that if the doctors would only advertise more extensively and let people know who and what they are that the quack's harvest would be materially reduced.

Now, one word in regard to the code of ethics. If I understand it rightly, it is simply to ensure professional courtesy and for mutual protection. Such a code is desirable and commendable, but that often practiced bears as much resemblance to the true code as a dude does to a gentleman.

Let me in conclusion say—and say honestly—that as a body I honor the medical profession, appreciate its excellence and admire its charity. For years I have been on intimate association with doctors, some of my dearest friends are in their ranks and even I—at one time—"walked" St. Mary's Hospital, London, with a view to embracing the profession. Thus, knowing and sympathizing with both professions, I have dared to write freely and honestly, laying bare the disease in order that it may be fully examined, and a radical cure effected. I have not done this to abuse, ridicule or injure the profession, but in order that the doctors may clearly understand the newspaper men's views, and that, understanding them, a better and more amicable spirit may be engendered between the doctors and the newspaper men for the common good.

